# The Pafah1b Complex Interacts with the Reelin Receptor VLDLR

**DOI:** 10.1371/journal.pone.0000252

**Published:** 2007-02-28

**Authors:** Guangcheng Zhang, Amir H. Assadi, Robert S. McNeil, Uwe Beffert, Anthony Wynshaw-Boris, Joachim Herz, Gary D. Clark, Gabriella D'Arcangelo

**Affiliations:** The Cain Foundation Laboratories, Texas Children's Hospital, Houston, Texas, United States of America; Department of Pediatrics, Baylor College of Medicine, Houston, Texas, United States of America; Department of Neuroscience, Baylor College of Medicine, Houston, Texas, United States of America; Department of Neurology, Baylor College of Medicine, Houston, Texas, United States of America; Program in Developmental Biology, Baylor College of Medicine, Houston, Texas, United States of America; Program in Translational Biology and Molecular Medicine, Baylor College of Medicine, Houston, Texas, United States of America; Department of Molecular Genetics, University of Texas Southwestern Medical Center, Dallas, Texas, United States of America; Department of Pediatrics and Medicine, University of California, San Diego School of Medicine, La Jolla, California, United States of America; University of Giessen, Germany

## Abstract

Reelin is an extracellular protein that directs the organization of cortical structures of the brain through the activation of two receptors, the very low-density lipoprotein receptor (VLDLR) and the apolipoprotein E receptor 2 (ApoER2), and the phosphorylation of Disabled-1 (Dab1). Lis1, the product of the *Pafah1b1* gene, is a component of the brain platelet-activating factor acetylhydrolase 1b (Pafah1b) complex, and binds to phosphorylated Dab1 in response to Reelin. Here we investigated the involvement of the whole Pafah1b complex in Reelin signaling and cortical layer formation and found that catalytic subunits of the Pafah1b complex, Pafah1b2 and Pafah1b3, specifically bind to the NPxYL sequence of VLDLR, but not to ApoER2. Compound *Pafah1b1^+/−^*;*Apoer2^−/−^* mutant mice exhibit a *reeler*-like phenotype in the forebrain consisting of the inversion of cortical layers and hippocampal disorganization, whereas double *Pafah1b1^+/−^*;*Vldlr^−/−^* mutants do not. These results suggest that a cross-talk between the Pafah1b complex and Reelin occurs downstream of the VLDLR receptor.

## Introduction

Heterozygous mutations in the *PAFAH1B1* (*LIS1*) gene in humans causes a reduction in the number of cortical gyri (lissencephaly) [1]. Homozygous mutations in the *REELIN* (*RELN*) gene also result in lissencephaly, with additional cerebellar hypoplasia [2]. Reelin, a secreted glycoprotein controlling neuronal positioning, functions by clustering its receptors VLDLR and ApoER2, causing the activation of src-family kinases (SRKs) and the phosphorylation of the adapter molecule Dab1 (reviewed by [3]–[5]). Disruption of *Reelin* (*Reln*) in homozygous *reeler* mice results in cortical layer disruption, cerebellar hypoplasia and ataxia. Mice deficient for Dab1 [6]–[8], both VLDLR and ApoER2 receptors [9] and SFKs Fyn and Src [10] exhibit a *reeler*-like phenotype.

Homozygous deletions of the *Pafah1b1* gene (encoding Lis1) in the mouse result in early embryonic lethality, whereas heterozygous mutations lead to hippocampal lamination defects [11]. Further reduction of Lis1 activity in compound hypomorphic mutants led to the disruption of cortical layers [12]. Lis1 was initially identified as the non-catalytic β subunit of the Pafah1b complex [1], [13]. This complex also contains two catalytic α subunits encoded by the *Pafah1b2* and *Pafah1b3* genes that hydrolyze the platelet-activating factor [14]. The product of the *Pafah1b2* gene (α2) is 30 kDa, whereas the product of the *Pafah1b3* gene (α1) is a 29 kDa protein. The entire Pafah1b complex resembles a G-protein signaling complex [15], [16]. In the mouse, mutations in the α subunit genes cause no overt neurological phenotype, but loss of Pafah1b2 disrupts spermatogenesis [16], [17]. In humans, *PAFAH1B3* hemizygousity is associated with mental retardation and ataxia [18], suggesting that the catalytic subunits of Pafah1b may be important for brain development or function. In addition to its involvement with the Pafah1b complex, Lis1 participates in cytoskeletal dynamics as a component of an evolutionary conserved pathway that mediates nucleokinesis (reviewed by [19]). Lis1 regulates the function of cytoplasmic dynein/dynactin motor complex [20], [21] through binding to several of its components. These interactions are thought to be important for several aspects of brain development, including neural stem cell proliferation and neuronal migration [22].

We previously showed that Lis1 binds Dab1 in a Reelin-dependent manner and that Lis1 and Reelin functionally interact [23]. Given the association of Lis1 with the Pafah1b α subunits, we investigated the interaction between this complex and the Reelin pathway. Here we demonstrated that both α subunits bind the Reelin receptor VLDLR but not ApoER2. Genetic experiments further demonstrated that loss of ApoER2 combined with Lis1 reduction results in a *reeler*-like phenotype, suggesting that the Pafah1b complex modulates the Reelin pathway.

## Results

### Pafah1b3 and Pafah1b2 bind to VLDLR but not to ApoER2

**Figure 1:**
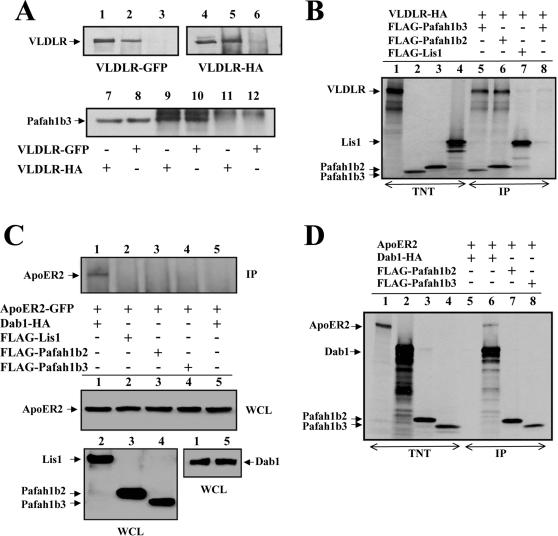
Pafah1b2 and Pafah1b3 bind VLDLR but not ApoER2. (A) Pafah1b3 binds VLDLR in transfected cells. Pafah1b3-MYC was co-expressed in 293T cells with GFP- (lanes 1–3) or HA-tagged VLDLR (lanes 4–6). VLDLR was co-precipitated with MYC antibodies directed against Pafah1b3 (lanes 2 and 5), but not with control antibodies (lanes 3 and 6). Conversely, Pafah1b3 was co-precipitated with HA (lane 9) or GFP (lane 10) directed against VLDLR, but not with control antibodies (lanes 11–12). Lanes 1 and 4 show VLDLR, and lanes 7–8 show Pafah1b3 in the WCL. Blots were probed with the GFP (lanes 1–3), HA (lanes 4–6) or Myc antibodies (lanes 7–12). (B) Pafah1b2 and Pafah1b3 bind VLDLR in a cell-free-system (TNT). *In vitro* translated proteins were radiolabeled with ^35^S and analyzed by SDS-PAGE (lanes 1–4). Individual Pafah1b subunits were combined with equal amounts of VLDLR and immunoprecipitated with FLAG (lanes 5–7) or a negative control antibody (lanes 8). Proteins were detected by autoradiography. Note that VLDLR co-precipitated with Pafah1b α subunits, but not with Lis1. (C) Pafah1b2 and Pafah1b3 do not bind ApoER2 in transfected cells. ApoER2-GFP was co-expressed in 293T cells with the indicated proteins, and co-immunoprecipitated with HA antibodies directed against Dab1 (lane 1). FLAG antibodies directed against the Pafah1b subunits (lanes 2–4) or a control antibody (lane 5) did not co-precipitate the receptor. Blots were probed with antibodies against GFP to detect co-precipitated ApoER2 (upper panel) or total ApoER2 expression in the corresponding WCLs (middle panel). FLAG or HA antibodies were used to detect Pafah1b subunits (lower left panel) or Dab1 (lower right panel) in the corresponding WCLs. ApoER2 co-precipitated exclusively with Dab1. (D) Pafah1b2 and Pafah1b3 do not bind ApoER2 in a cell-free-system (TNT). *In vitro* translated proteins were radiolabeled with ^35^S and analyzed by SDS-PAGE (lanes 1–4). Equal amounts of ApoER2 were combined with the indicated proteins and immunoprecipitated with a control antibody (lane 5), or with antibodies against Dab1 (lane 6), Pafah1b2 (lane 7) or Pafah1b3 (lane 8). Proteins were detected by autoradiography. ApoER2 again co-precipitated only with Dab1. IP, immunoprecipitate; WCL, whole cell lysate.

Previous observations revealed the genetic interaction of *Pafah1b1* with the genes encoding components of the *Reln* signaling pathway, and the direct binding of Lis1 to phosphorylated Dab1 [23]. In this study we turned our attention to the catalytic α subunits of the Pafah1b complex. To investigate potential biochemical interactions of Pafah1b α subunits with the Reelin receptors VLDLR and ApoER2, co-immunoprecipitation assays were performed. Plasmids encoding Pafah1b3 (tagged with a MYC epitope) and VLDLR (tagged with either GFP or HA epitopes) were transfected in 293T cells. Results show that VLDLR was co-immunoprecipitated with antibodies directed against the Pafah1b3 tag and, conversely, that Pafah1b3 was co-immunoprecipitated with antibodies directed against the VLDLR tag (Fig. 1A). To determine whether this binding was direct, individual FLAG-tagged Pafah1b subunits were expressed in a cell-free system and incubated in the presence of *in vitro*- translated VLDLR. The receptor was efficiently co-immunoprecipitated with either Pafah1b2 or Pafah1b3, but not with Lis1 (Fig. 1B), indicating that the α subunits are capable of binding VLDLR directly.

Similar experiments were performed to determine whether Pafah1b3 and Pafah1b2 interact with ApoER2. When this receptor was co-expressed with either Pafah1b2 or Pafah1b3 in 293T cells, immunoprecipitation experiments failed to reveal any interaction (Fig. 1C). As a positive control we used Dab1, which is known to bind lipoprotein receptors [9]. Immunoprecipitation assays using *in vitro* translated proteins also revealed no interaction between α subunits and ApoER2, whereas Dab1 was able to bind the receptor, as expected (Fig. 1D). Together, these results demonstrate that both Pafah1b2 and Pafah1b3 α subunits interact specifically with the Reelin receptor VLDLR, but not with ApoER2.

**Figure 2:**
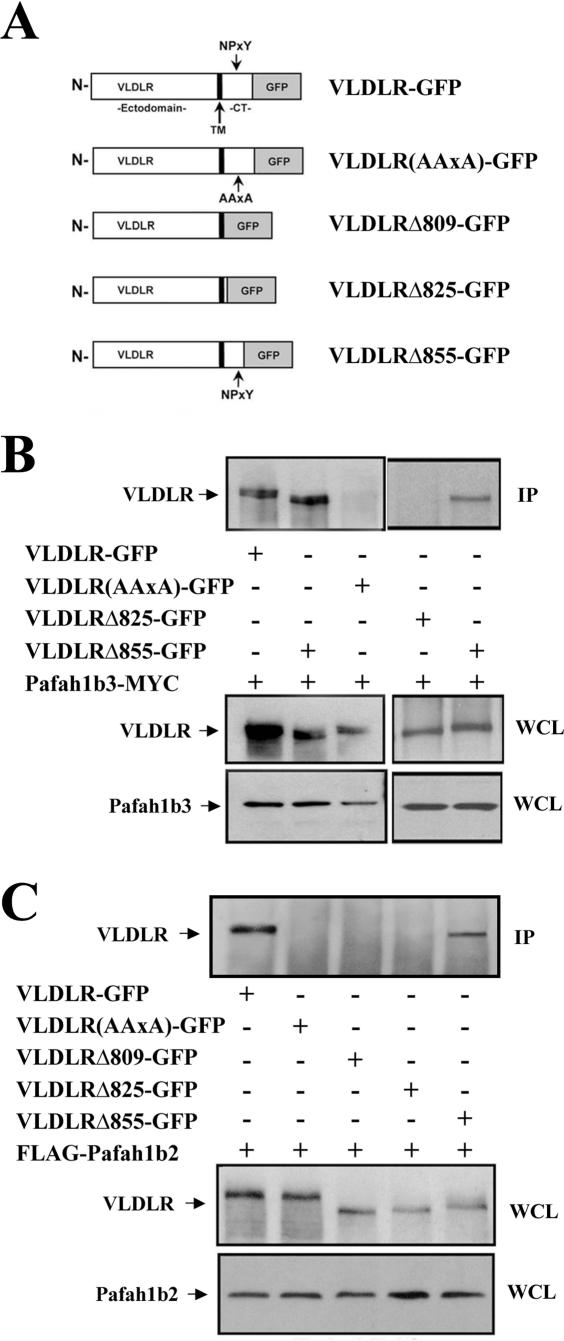
Pafah1b2 and Pafah1b3 interact specifically with the NPxY motif of VLDLR. (A) Diagram of the VLDLR expression constructs used in this study. TM = transmembrane region. CT = cytoplasmic tail. (B) The NPxY motif is required for VLDLR binding to Pafah1b3. Pafah1b3-MYC was co-expressed in 293T cells with the indicated GFP-tagged VLDLR constructs. Proteins were immunoprecipitated with Myc antibodies and the blot was probed with GFP antibodies to detect VLDLR receptors (upper panel). Only full-length VLDLR and the NPxY-containingVLDLR?855 co-precipitated with Pafah1b3. WCLs were probed with GFP (middle panels) or Myc (lower panels) antibodies to ensure that similar amounts of VLDLR or Pafah1b3 proteins were present in each sample. (C) The NPxY motif is required for VLDLR binding to Pafah1b2. FLAG-Pafah1b2 was co-expressed in 293T cells with GFP-tagged VLDLR constructs and immunoprecipitated with FLAG antibodies. Blots were probed with GFP antibodies to detect VLDLR proteins in the IP (upper panel) or WCLs (middle panel), or FLAG antibodies to detect Pafah1b2 in the WCLs (lower panel). Only full-length VLDLR and VLDLR?855 co-precipitated with Pafah1b2. IP, immunoprecipitate; WCL, whole cell lysate.

The cytoplasmic NPxY domain of lipoprotein receptors enables binding of proteins containing PTB domains such as Dab1 [9]. To determine whether the NPxY motif is required for Pafah1b2 and Pafah1b3 binding to VLDLR, a series of C terminal truncation mutants of the receptor tagged with GFP were produced (Fig. 2A). Co-immunoprecipitation experiments were conducted in COS7 cells using antibodies against the α subunit tag. The VLDLR ectodomain fragment (VLDLRΔ809) and the C-terminal truncation mutant VLDLRΔ825, both lacking the NPxY domain, showed no interaction with Pafah1b3 (Fig. 2B) or with Pafah1b2 (Fig. 2D). On the other hand, the truncation construct VLDLRΔ855 that retained the NPxY motif did exhibit binding to Pafah1b3 (Fig. 2B and C) and to Pafah1b2 (Fig. 2D). Finally, the specific NPxY mutant, VLDLR(AAxA), showed loss of interaction with either Pafah1b3 (Fig. 2C) and with Pafah1b2 (Fig. 2D). These results demonstrated that α subunit binding to VLDLR requires the presence of an intact NPxY motif.

**Figure 3:**
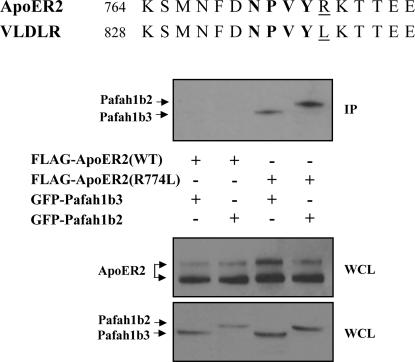
Substitution of Arg^774^ with a Leu in ApoER2 rescues binding to Pafah1b α subunits. (A) Amino acid sequence of VLDLR and ApoER2 near the NPxY motif in the cytoplasmic tail of each receptor. Unique amino acids R774 in ApoER2 and L838 in VLDLR are underlined. (B) A Leu residue following the NPxY motif is required for Pafah1b α subunit binding to lipoprotein receptors. FLAG-tagged intact ApoER2(WT) or a mutant receptor in which Arg^774^ was substituted by a Leu ApoER2(R774L) were co-expressed in COS7 cells with GFP-tagged Pafah1b α subunits. Proteins were immunoprecipitated with FLAG antibodies (upper panel) and immunoblotted with antibodies against GFP to detect co-precipitated α subunits. FLAG (middle panel) or GFP (lower panel) antibodies were used to detect ApoER2 receptors or the Pafah1b α subunits in the WCL. Pafah1b2 and Pafah1b2 co-precipitated with ApoER2(R774L), but not with intact ApoER2(WT). IP, immunoprecipitate; WCL, whole cell lysate.

The cytoplasmic regions of VLDLR and ApoER2 that include the NPxY binding domain possess significant sequence homology, yet we found that Pafah1b2 and Pafah1b3 bind only to VLDLR. To understand this apparent discrepancy, we examined the protein sequence of the receptor intracellular domains. The NPxY motif sequence in both receptors is NPVY, but the first amino acid downstream of this motif differs. In VLDLR this residues corresponds to a leucine (Leu^838^), whereas in ApoER2 is an arginine (Arg^774^) (Fig. 3A). To determine whether a leucine at this position is important for Pafah1b2 and Pafah1b3 binding, an ApoER2 mutant receptor was generated in which Arg^774^ was substituted by a leucine (R774L). Co-immunoprecipitation experiments in transfected cells demonstrated that ApoER2 carrying the R774L mutation was able to bind Pafah1b2 and Pafah1b3, unlike the wild type receptor (Fig. 3B). These observations suggest that the NPVYL sequence of VLDLR mediates its unique property of binding to the Pafah1b α subunits. In contrast, Dab1 does not appear to discriminate between lipoprotein receptors as it binds to both, the NPVYL sequence of VLDLR and the NPVYR sequence of ApoER2.

**Figure 4:**
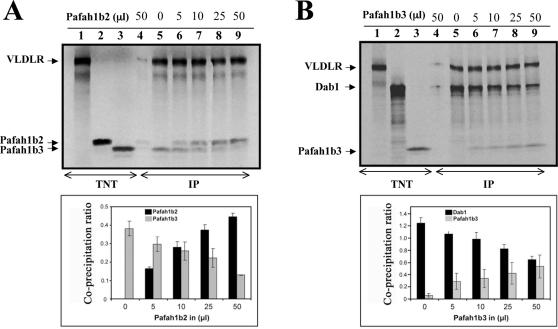
Pafah1b α subunits compete with each other and with Dab1 for binding to VLDLR. (A) Pafah1b α subunits compete for VLDLR binding. Proteins were expressed *in vitro* using a cell-free-system (TNT) and radiolabeled with ^35^S (lanes 1–3). Equal amounts (50 μl) of VLDLR and Pafah1b3 were incubated with increasing amounts of Pafah1b2, as indicated. Proteins were immunoprecipitated with polyclonal antibodies against VLDLR (lanes 5–9) or a control antibody (lane 4) and detected by autoradiography. The plot represents the mean ratio of co-precipitated Pafah1b3 and Pafah1b2 normalized to the amount of precipitated VLDLR in each sample. Increasing amounts of Pafah1b2 reduced the amount of co-precipitated Pafah1b3. (B) Proteins were expressed *in vitro* (TNT) and radiolabeled with ^35^S (lanes 1–3). Equal amounts (50 μl) of VLDLR and Dab1 were incubated with increasing amounts of Pafah1b3, as indicated. Proteins were immunoprecipitated with polyclonal antibodies against VLDLR (lanes 5–9) or a control antibody (lane 4) and detected by autoradiography. The plot represents the mean ratio of co-precipitated Pafah1b3 and Dab1 normalized to the amount of precipitated VLDLR in each sample. Increasing amounts of Pafah1b3 reduced the amount of co-precipitated Dab1. Bars represent the standard error of the mean from triplicate experiments. IP, immunoprecipitate.

Since the Pafah1b α subunits and Dab1 bind a similar region of VLDLR, we reasoned that they might compete for receptor occupancy. Indeed, increasing concentrations of Pafah1b2 were found to displace Pafah1b3 from VLDLR in co-immunoprecipitation experiments *in vitro* (Fig. 4A). Furthermore, increasing concentrations of Pafah1b3 were able to partially displace Dab1 from VLDLR, consistently with our observation that they bind similar residues of the receptor intracellular domain (Fig. 4B).

### In vivo analysis of the Pafah1b complex function in cortical lamination

**Figure 5:**
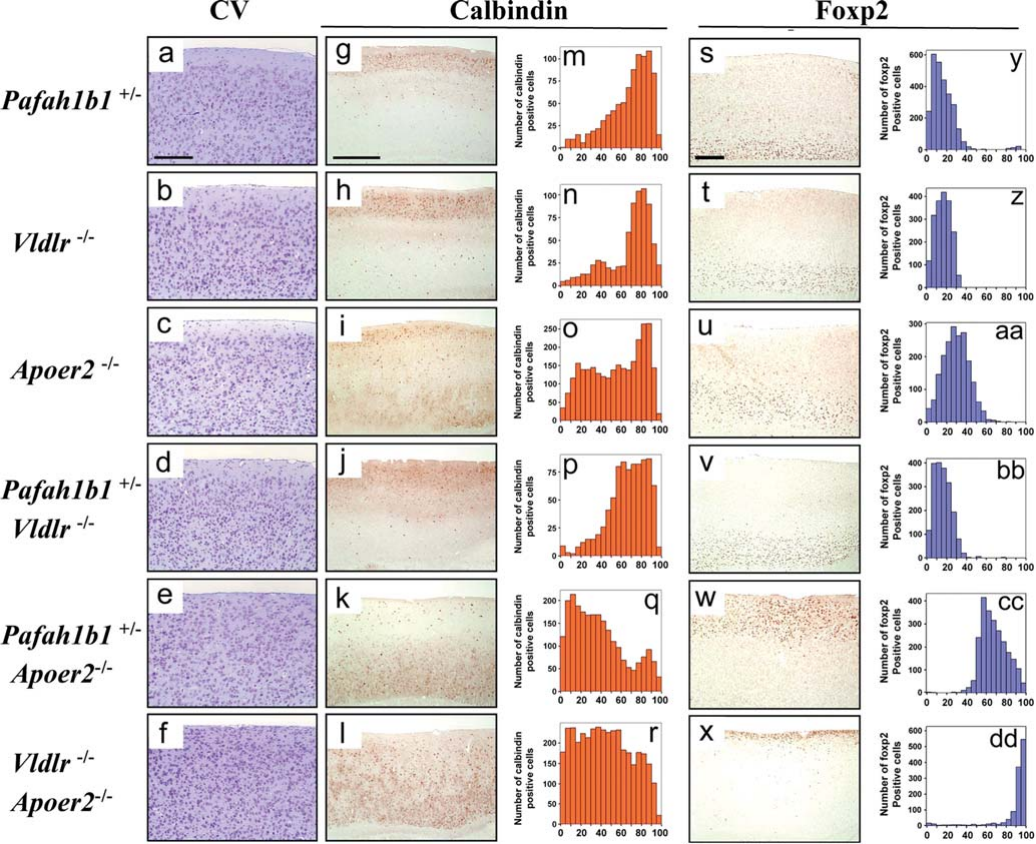
*reeler*-like disruption of cortical layers in *Pafah1b1 ^+/−^* mice lacking ApoER2 but not VLDLR. Sagittal sections of the neocortex were obtained from adult mice of the indicated genotype. Adjacent sections were stained with cresyl violet (CV) (a-f) or subjected to immunohistochemistry with antibodies against calbindin to label cells in layers II–III (g-l), or Foxp2 to label cells in layer VI (s-x). Histograms represent the radial distribution of cells positive for calbindin (m-r) or Foxp2 (y-dd) from the bottom of the cortical plate (set as 0) to the pial surface (set as 100). *Pafah1b1^+/−^* and *Vldlr ^−/−^* single or double mutants presented no obvious cortical layering defects. *Apoer2 ^−/−^* single mutants exhibited some laminar dispersion of upper layer neurons. In contrast, *Pafah1b1 ^+/−^;Apoer2 ^−/−^* double mutants displayed a marked inversions of upper and lower layers (*reeler*-like phenotype) comparable to that seen in *Apoer2 ^−/−^;Vldlr ^−/−^* mice. Scale bars = 500 µm.

The biochemical data presented above suggests that the α subunits may bring the whole Pafah1b complex in proximity of the VLDLR receptor. Since this receptor also binds Dab1, our findings raised the possibility that the Pafah1b complex may modulate Reelin signaling. To address this possibility *in vivo*, we set out to generate double mutant mice carrying mutations in genes encoding Pafah1b subunits as well as in genes encoding Reelin receptors. Given that both, *Apoer2* and *Pafah1b2* male knock out mice are sterile [16], [24], we could not generate double mutants that lacked these proteins. We were however able to readily generate compound mice that were heterozygous for *Pafah1b1* and homozygous for either *Apoer2* or *Vldlr*. Cortical lamination in these mutants was analyzed as a read-out of Reelin activity in brain development and compared to single mutants or to double *Apoer2*/*Vldlr* mutants, which exhibit a *reeler*-like phenotype. Cortical sections were stained with two cellular layer-specific neuronal markers, Calbindin (layer II/III) and Foxp2 (layer VI) and the layer distribution of immunolabeled cells was analyzed quantitatively. This analysis revealed no obvious lamination defects in single *Pafah1b1* ^+/−^ or *Vldlr* ^−/−^ mice, whereas some layer abnormalities were observed in single *Apoer2* ^−/−^ mice, as previously reported [9], [25] (Fig. 5). The cortex of *Pafah1b1* ^+/−^;*Vldlr* ^−/−^ double mutants also appear fairly normal, however, that of *Pafah1b1* ^+/−^;*Apoer2* ^−/−^ double mutants presented a severe abnormality similar to the cortical layer inversion typically seen in *Reln* ^−/−^, *Dab1* ^−/−^ or *Apoer2* ^−/−^ *;Vldlr* ^−/−^ mutants (Fig. 5). Calbindin-positive neurons destined for upper layers were ectopically located in the lower cortex, whereas Foxp2-positive neurons destined for a lower layer were ectopically located into the upper cortex. The cortex of *Pafah1b1* ^+/−^;*Apoer2* ^−/−^ mutants also revealed hypercellularity of layer I, another typical feature of the *reeler* phenotype that is also seen in double *Apoer2* ^−/−^ *;Vldlr* ^−/−^ mutants (Fig. 5).

**Figure 6:**
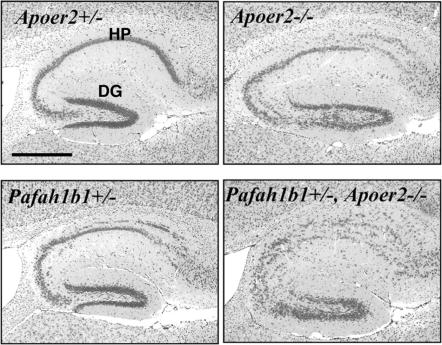
*reeler*-like disruption of hippocampal layers in *Pafah1b1 ^+/−^*;*Apoer2^−/−^* double mutants. Comparable sagittal sections of the hippocampus obtained from adult mice of the indicated genotype were stained with cresyl violet. The hippocampus proper (HP) and the dentate gyrus (DG) of *Apoer2^+/−^* mice are normal, whereas a splitting of the pyramidal layers is evident in *Pafah1b1^+/−^* and in *Apoer2^−/−^* mice. Severe dyslamination of cellular layers is seen in double *Pafah1b1^+/−^*;*Apoer2^−/−^* (*reeler*-like). Scale bar = 500 µm.

To gain further evidence of the occurrence of a *reeler*-like phenotype in *Pafah1b1* ^+/−^;*Apoer2* ^−/−^ double mutants, we also examined the anatomy of hippocampal structures (Fig. 6). Cellular layers in the hippocampus proper and dentate gyrus were normal in heterozygous *Apoer2* ^+/−^ mice, whereas a modest split of the pyramidal layer in area CA1 and CA3 was observed in *Pafah1b1* ^+/−^ and homozygous *Apoer2* ^−/−^ mice. However, a *reeler*-like phenotype characterized by profound dyslamination of all cellular layers was observed in double *Pafah1b1* ^+/−^;*Apoer2* ^−/−^ mice (Fig. 6). No gross abnormalities were observed in the cerebellum of *Pafah1b1* ^+/−^;*Apoer2* ^−/−^ double mutants, unlike *reeler* mice which exhibit profound cerebellar hypoplasia (not shown). Together, these data demonstrate that *Pafah1b1*, like *Vldlr* mutations, synergize with *Apoer2* disruption and contribute to the appearance of a *reeler*-like phenotype, at least in the forebrain.

### Reelin signaling is largely intact in Pafah1b1^+/−^; Apoer2^−/−^ cortical neurons

**Figure 7:**
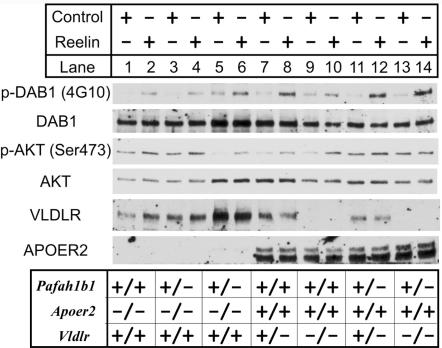
Reelin induces Dab1 and Akt phosphorylation in *Pafah1b1 ^+/−^*;*Apoer2^−/−^* double mutant neurons. Cortical neurons were cultured from mutant mice of the indicated genotype, and incubated with either control or Reelin-containing medium for 20 min. Lysates were analyzed by Western blot using the 4G10 antibody to detect Dab1 phosphorylation on tyrosine residues and a phospho-Akt antibody to detect Akt phosphorylation on serine 473. Blots were reprobed with antibodies against total Dab1 and Akt to ensure that similar amount of proteins were present in each sample, and with antibodies against VLDLR and ApoER2 to confirm the genotype of the mutants.

In normal neurons, Reelin treatment induces Dab1 phosphorylation on tyrosine residues [26]–[28] and the activation of PI3K, which leads to the phosphorylation of Akt on serine residue 473 [29]–[31]. In double *Vldlr^−/−^;Apoer2^−/−^* mutant mice the appearance of the anatomical phenotype correlates with loss of these Reelin-dependent signaling events [29]. Since double *Pafah1b1^+/−^; Apoer2* ^−/−^ mutants exhibit a *reeler*-like phenotype in the forebrain, we sought to determine whether Reelin signaling was also affected. Cortical neurons were obtained from mice carrying *Pafah1b1, Apoer2* and *Vldlr* mutations, alone or in combination. As for normal mice, Reelin treatment was found to induce Dab1 and Akt phosphorylation in all mutants examined, including *Pafah1b1* ^+/−^ *;Apoer2* ^−/−^ mutants, even though these animals exhibit cortical layers defects (Fig. 7). These data indicate Dab1 and Akt phosphorylation is not sufficient to induce cortical layer formation.

## Discussion

We have previously demonstrated the existence of an interaction between Reelin and Lis1 signaling consisting of the direct binding of Lis1, the regulatory subunit of the Pafah1b complex, to the adapter Dab1 [23]. This interaction takes place when Dab1 is phosphorylated on tyrosine residues in response to Reelin. In the present study we have examined the interaction of individual Reelin receptors with the subunits of the Pafah1b complex. We shown that the catalytic α subunits of the Pafah1b complex, Pafah1b2 and Pafah1b3, bind specifically VLDLR and that a reduction in Lis1 activity mimics the loss of this receptor in the forebrain. The binding of Pafah1b3 and Pafah1b2 to VLDLR requires the NPxY domain and the presence of a leucine residue immediately following this sequence. The catalytic α subunits cannot bind the NPxYR sequence of ApoER2, but a point mutation that converts the arginine residue adjacent to the NPxY motif to a leucine rescued Pafah1b α subunit binding, demonstrating that this residue is critical for coupling the Pafah1b complex selectively with VLDLR. Given the low abundance of this receptor in neurons, we could not confirm that the interactions we observed in transfected cells and *in vitro* also take place in normal neurons. However, given the specificity of the Pafah1b α subunits for VLDLR and the strict requirement for the NPVYL sequence, it seems reasonable to conclude that the binding may indeed occur *in vivo*.

**Figure 8:**
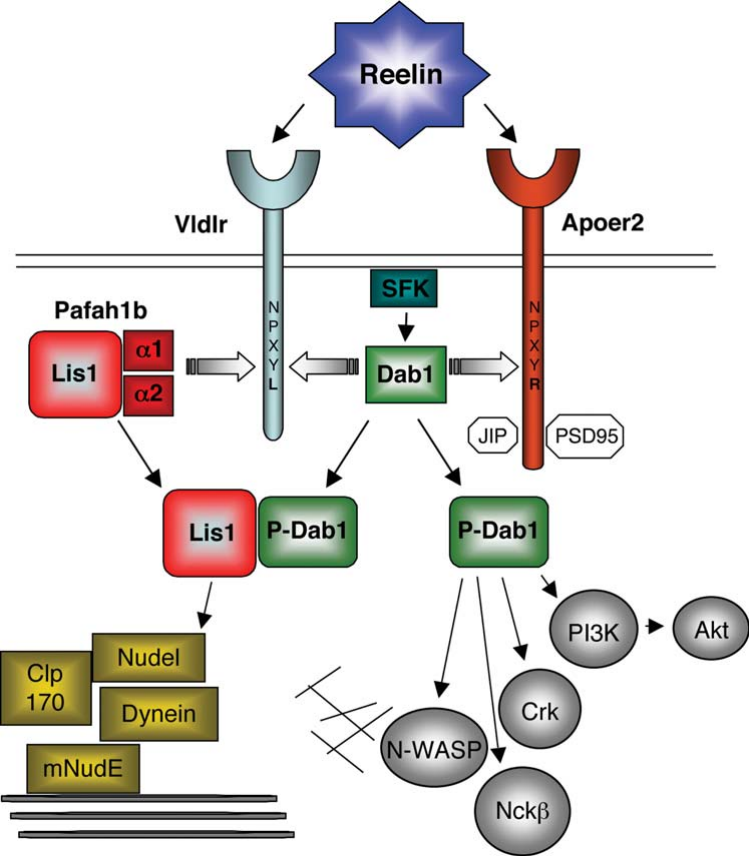
Integrated model of Reelin and Lis1 signaling. Reelin binds to VLDLR and ApoER2 and causes src-family kinase (SFK) activation and Dab1 phosphorylation. Dab1 binds to the NPxY motif of both, VLDLR and ApoER2. Upon Reelin stimulation, phosphoDab1 (P-Dab1) interacts with Lis1 and with other signal transduction molecules (grey circles). Lis1 also binds the catalytic subunits of the Pafah1b complex (α1 and α2) as well as components of the cytoplasmic dynein complex (yellow square). α1 and α2 also bind VLDLR at the NPxYL motif and compete with Dab1 for receptor occupancy. These subunits do not recognize the NPxYR motif of ApoER2. A unique domain of ApoER2 enables unique interactions with synaptic and trafficking proteins (white octagons). The binding of catalytic Pafah1b subunits to VLDLR may displace P-Dab1 and promote its interaction with Lis1. Signaling molecules downstream of Lis1 and Dab1 affect cytoskeleton dynamics by acting on microtubules (thick lines) or actin filaments (thin lines), thereby controlling neuronal migration and layer formation.

Through our genetic studies, we demonstrated that the biochemical interaction of the Pafah1b complex with VLDLR has physiological consequences for forebrain development. Consistent with our biochemical data, we observed that *Pafah1b1^+/−^* mutations had no effect on the appearance of brain structures in *Vldlr^−/−^* mutants, suggesting that the products of these genes may function in a linear pathway. On the other hand *Pafah1b1^+/−^* mutations exacerbated the phenotype of *Apoer2^−/−^* mutants to an extent that the appearance of cortical and hippocampal structures in double *Pafah1b1^+/−^*; *Apoer2^−/−^* mutants resembled that of *reeler* mice. Since a *reeler*-like phenotype is also observed in double *Vldlr^−/−^*; *Apoer2^−/−^* mutants [9], these data suggest that Lis1 is an important component of the Reelin signaling pathway downstream of VLDLR (Fig. 8). The simplest interpretation of our data is that the α subunits function as signaling adapter molecules by bringing Lis1 in proximity of the VLDLR receptor and Dab1, thus facilitating Reelin signaling through this receptor. An alternative interpretation of our genetic findings is that ApoER2 is the dominant Reelin receptor in forebrain development, and that the consequences of *Pafah1b1^+/−^* mutations on Reelin signaling can only be appreciated when this receptor is missing. This view is supported by the observation that *Apoer2^−/−^* mutations in isolation already result in a noticeable cortical and hippocampal phenotype, unlike *Vldlr^−/−^* mutations [9], [25]. Both interpretations of our data are consistent with a functional role for the Pafah1b complex in Reelin signaling during brain development.

Despite the disorganization of cortical layers, we observed that the induction of Dab1 and Akt phosphorylation by Reelin was fairly normal in neurons isolated from *Pafah1b1^+/−^*; *Apoer2^−/−^* double mutant brains. This is in striking contrast to the results obtained using *Vldlr^−/−^*; *Apoer2^−/−^* double mutant neurons, in which Reelin does not appear capable to elicit any signaling events [25], [29]. Similarly, Reelin treatment did not induce Akt phosphorylation in *Dab1* ^−/−^ neurons [29], [31]. Our findings indicate that the Pafah1b complex and Lis1 are not required for many of the signaling events which are normally stimulated by Reelin mainly through clustering of the ApoER2 receptor. In the absence of ApoER2, Reelin signaling events such as Dab1 and Akt phosphorylation still occur, albeit to a lower level, mediated by the VLDLR receptor and irrespective of the presence of Pafah1b proteins. However, under these reduced signaling conditions, Lis1 deficiency prevents the formation of cortical layers. Together with our previous observation that Lis1 binds exclusively phospho-Dab1 [23], the present findings suggest that Lis1 functions downstream of SFK activity and it is not predicted to interfere with the interaction between Dab1 and other signaling molecules such as PI3K [29]–[31], Nckβ [32], Crk family proteins [28], [33], or N-WASP [34].

We and others have previously shown that loss of Pafah1b α subunits in the mouse does not result in a neurological phenotype, but affects spermatogenesis [16], [17]. These studies indicate that the catalytic subunits of the Pafah1b complex are not absolutely required for brain development. Based on the present data we propose that they may modulate Reelin signaling downstream of VLDLR, possibly by promoting Lis1 and Dab1 interaction.

VLDLR and ApoER2 are both individually capable of binding Reelin on the extracellular side and Dab1 on the intracellular side, and both contribute to cortical layer formation [9], [25], [35], [36]. In addition, ApoER2 is known to bind JNK Interacting Protein (JIP) 1 and 2 and PSD-95 through a unique intracellular domain encoded by an alternatively spliced exon [37]–[39]. Recent studies demonstrated that ApoER2 interacts with the NMDA receptor, thereby mediating a Reelin-dependent function in learning and memory in the adult brain [40]. Here we have shown that VLDLR is also capable of unique interactions that may affect Reelin signaling and forebrain cellular layer formation. It has recently been reported that *VLDLR* deletions in humans result in a neurological disorder characterized by lissencephaly and cerebellar hypoplasia, malformations similar but less severe than those associated with *RELN* deletions [41]. Thus, in humans an overt neurological phenotype is seen even in the presence of the ApoER2, further underscoring the importance of VLDLR in brain development. It remains to be determined whether the unique ability of VLDLR to bind the Pafah1b complex affects postnatal brain function in addition to neuronal positioning during embryogenesis.

## Materials and Methods

### Generation of plasmid constructs

Human *VLDLR* cDNA encoding the 873 amino acids isoform A (accession # NP_003374) was cloned into pEGFP-N1 (Clontech) to generate the VLDLR-GFP fusion protein. Alternatively, the same cDNA was tagged at the C terminus with the HA epitope by PCR. Truncation constructs VLDLRΔ809-GFP (containing VLDLR amino acids 1-809), VLDLRΔ825-GFP (containing VLDLR amino acids 1-825) and VLDLRΔ855-GFP (containing VLDLR amino acids 1-855) were generated using the Expand High Fidelity PCR System. To produce VLDLR(AAxA)-GFP, site directed mutagenesis was performed on the VLDLR-GFP plasmid using the Stratagene QuickChange Mutagenesis Kit. Mouse *Apoer2* cDNA (a gift from J. Nimpf, Medical University of Vienna, Austria) encoding the full-length receptor (accession # CAC38356) minus the alternatively spliced 59 amino acids exon 19 was cloned in frame with GFP or HA as described above for VLDLR. The FLAG-ApoER2(WT) construct was generated by subcloning ApoER2-HA into pCMV-Tag (Stratagene). This construct was further mutagenized to generate the FLAG- ApoER2(R774L) in which Arg residue 774 is replaced by a Leu. All constructs were sequenced to verify the intended mutations. Mouse *Dab1* cDNA (accession # NP_796233) encoding the 555 amino acids isoform 2 was HA-tagged by PCR and subcloned into pcDNA3.1 vector (Invitrogen). Mouse *Pafah1b3* cDNA (accession #Q61205) encoding the 29 kDa α1 subunit, mouse *Pafah1b2* cDNA (accession # Q61206) encoding the 30 kDa α2 subunit, and mouse *Pafah1b1* cDNA (accession #NP_038653) encoding the 45 kDa β1 subunit of the Pafah1b complex, were cloned into the pEGFP-C1 (Clontech), pcDNA3.1(+)-myc/his (Invitrogen) or pCMV-Tag (Stratagene) to introduce the GFP, Myc or FLAG tag, respectively.

### Cell culture and protein analysis

COS7 or 293T cells (ATCC) were cultured in DMEM supplemented with 10% fetal bovine serum, and transfected with expression vectors using the Fugene 6 reagent (Roche). After 30–40 hours, the cells were harvested and the proteins extracted in lysis buffer (PBS, 5 mM EDTA, 1% Triton X-100, pH 7.4) in the presence of protease inhibitors (Mini Complete protease inhibitor cocktail tablets, Roche). For immunoprecipitation, the lysates were incubated with appropriate antibodies for 1–2 hours at 4°C, followed by protein A/G agarose beads (Pierce). Samples were analyzed by SDS-PAGE. To assay Reelin signaling, primary cortical neurons were cultured from embryonic mice and treated with Reelin-containing conditioned medium for 20 min. Cells were lysed and proteins were subjected to Western blot analysis as previously described [29].

### In vitro binding assay

*Vldlr, Apoer2, Pafah1b1, Pafah1b3, Pafah1b2,* and *Dab1* cDNAs were produced *in vitro* using the TNT Quick Coupled Transcription/Translation System (Promega), according to the manufacturer instructions using ^35^S-labeled methionine (Amersham Biosciences). Proteins were separated by SDS-PAGE and detected by autoradiography on dried gels. Quantitative analysis of autoradiography bands density was performed using ImageJ software (NIH image).

### Animals

*Reeler* mutant mice were obtained from The Jackson Laboratories on a C57BL/6×C3H hybrid background. *Vldlr, Apoer2* and *Dab1* knock out mice were on a hybrid C57BL/6×129S6/SvEv. *Pafah1b1* (*Pafah1b1^neo^*, a null, was utilized in these studies) [11] was on a 129S6/SvEv background. Mutants were genotyped by PCR as described previously for *Pafah1b1* [11], *Reelin* [42], *Apoer2* and *Vldlr* [9], and *Dab1* [6].

### Quantification and statistical analysis of cortical sections

Sagittal paraffin sections (5 µm) of the brain from siblings (when possible) of each genotype were stained with cresyl violet or processed for immunohistochemistry using antibodies against Calbindin (layer II–III) or Foxp2 (layer VI). Four paramedian sagittal images at a level caudal to the corpus callosum were utilized for quantitative analysis as previously described [23].
